# The Treatment by Drugs

**Published:** 1892-12-17

**Authors:** 


### The Treatment of Rickets.

### The Treatment by Drugs.

The most successful drug, and the one most used at Great Ormond Street in the treatment of rickets is cod-liver oil. It is given in doses ranging from ʒss—ʒiii, according to the age of the child. It is generally advisable to give some preparation of iron, and the combination of the cod-liver oil with vinum ferri is a most valuable one. The syrup of the phosphate or iodide of iron often gives good results. The hypophosphites, and especially the lactophosphates, are most useful adjuncts to the dietetic treatment.

Phosphorus in doses of $\frac{1}{100}$ grain has not given very satisfactory results in the majority of cases in which it has been tried.

